# SDA 7: A modular and parallel implementation of the simulation of diffusional association software

**DOI:** 10.1002/jcc.23971

**Published:** 2015-06-29

**Authors:** Michael Martinez, Neil J. Bruce, Julia Romanowska, Daria B. Kokh, Musa Ozboyaci, Xiaofeng Yu, Mehmet Ali Öztürk, Stefan Richter, Rebecca C. Wade

**Affiliations:** ^1^ Molecular and Cellular Modeling Group, Heidelberg Institute for Theoretical Studies (HITS)Schloss‐Wolfsbrunnenweg 3569118HeidelbergGermany; ^2^ Heidelberg Graduate School of Mathematical and Computational Methods for the Sciences (HGS MathComp)Im Neuenheimer Feld 36869120HeidelbergGermany; ^3^ Hartmut Hoffmann‐Berling International Graduate School of Molecular and Cellular Biology (HBIGS)Im Neuenheimer Feld 50169120HeidelbergGermany; ^4^ Zentrum für Molekulare Biologie der Universität Heidelberg (ZMBH), DKFZ‐ZMBH AllianceIm Neuenheimer Feld 28269120HeidelbergGermany; ^5^ Interdisciplinary Center for Scientific Computing (IWR), Heidelberg UniversityIm Neuenheimer Feld 36869120HeidelbergGermany; ^6^ Department of Global Public Health and Primary CareUniversity of BergenKalfarveien 315018BergenNorway

**Keywords:** Brownian dynamics, protein flexibility, macromolecular association, biomacromolecular diffusion, parallelization, protein‐solid state interactions, protein adsorption

## Abstract

The simulation of diffusional association (SDA) Brownian dynamics software package has been widely used in the study of biomacromolecular association. Initially developed to calculate bimolecular protein–protein association rate constants, it has since been extended to study electron transfer rates, to predict the structures of biomacromolecular complexes, to investigate the adsorption of proteins to inorganic surfaces, and to simulate the dynamics of large systems containing many biomacromolecular solutes, allowing the study of concentration‐dependent effects. These extensions have led to a number of divergent versions of the software. In this article, we report the development of the latest version of the software (SDA 7). This release was developed to consolidate the existing codes into a single framework, while improving the parallelization of the code to better exploit modern multicore shared memory computer architectures. It is built using a modular object‐oriented programming scheme, to allow for easy maintenance and extension of the software, and includes new features, such as adding flexible solute representations. We discuss a number of application examples, which describe some of the methods available in the release, and provide benchmarking data to demonstrate the parallel performance. © 2015 The Authors. Journal of Computational Chemistry Published by Wiley Periodicals, Inc.

## Introduction

Protein–protein diffusional association processes can occur on timescales extending to the order of seconds, beyond the scope of current all‐atom molecular dynamics (MD) simulations. This problem, and the need to sample many molecular encounters to provide reliable statistics on association kinetics, mean that simplified models are required. One approach is to model protein diffusion by Brownian dynamics (BD) simulation of rigid solute bodies in a continuum model of the solvent. Apart from eliminating the solvent's degrees of freedom, the use of rigid solutes reduces the computational cost of simulations in two main ways. First, by neglecting high frequency intramolecular vibrations, a much larger simulation time step (on the order of picoseconds, compared to the femtosecond time steps required in MD simulations) may be used, greatly reducing the number of force evaluations required to propagate the simulation trajectory. Second, intermolecular interactions can be modeled as the interaction of fixed interaction sites on one solute with a precomputed interaction potential grid on another solute, reducing the formal scaling of the calculation of intermolecular forces to *O*(*N*), with *N* the number of solute atoms, compared to the $O(N^{2})$ or O(N ln N) algorithms in MD. This treatment of forces between solutes is used in simulation of diffusional association (SDA) and is also employed in other BD simulation software packages, namely, UHBD,^1^, ^2^ Browndye,^3^ BrownMove,^4^ and Macrodox.^5^

SDA is a BD simulation software package that was first reported in 1997 by Gabdoulline and Wade^6^, ^7^ for computing bimolecular rate constants for protein–protein association. The software was later extended to allow the study of protein–protein docking,^8^ electron transfer,^9^ and docking to a metal surface.^10^ More recently, Mereghetti et al.^11^ developed a variant of SDA (SDAMM) which allows the simulation of multiple macromolecules, to compute diffusional and associational properties, and investigate the effects of macromolecular crowding which occur at high macromolecular concentrations.^12^ Applications of SDA include studies of the effects of viral RNA binding on capsid protein association rates,^13^ the association of a soluble protein to a membrane bound protein,^14^ protein association to membrane phosphoinositide lipids,^15^ protein binding to cellulose fibres,^16^ and the characterisation of drug‐receptor encounter complexes.^17^ A BD method for simulating many solutes,^18^ based on SDA but developed independently, was also used to simulate a model of the bacterial cytoplasm.^19^

In this article, we report an updated and restructured version of SDA, SDA 7. Our motivation for creating this new version was driven by the following five factors: (i) our desire to unify the SDA and SDAMM codes, their associated preparation and analysis tools, and derived codes that model surface interactions, into a single maintainable framework; (ii) to improve the parallel performance of the software to better utilize modern parallel computer architectures; (iii) to introduce dynamic memory management to allow larger systems to be studied without the need for recompilation of the code; (iv) to allow solute flexibility to be treated in the simulated models; and (v) to improve the modularity and readability of the code to allow for easier maintenance and extension, both by developers and end‐users. In the sections that follow, we describe the theory of Brownian dynamics simulation of biomacromolecules and report the new features that have been developed as part of SDA 7, including a new algorithm which allows for solutes to be described by more than one rigid structure, thus incorporating conformational flexibility, or changes in protonation states, into the simulations. We then describe the new object‐oriented structure of the code, including a discussion of the reusability this adds to the framework, and describe the parallelization scheme employed. Finally, we give a set of simulation benchmarks and use cases.

## Brownian Dynamics Simulation of Macromolecular Diffusion

Modeling the interactions of biomacromolecules in solution is a highly complex problem with many degrees of freedom. One method to reduce the dimensionality of the problem is by using implicit solvent models in which the solvent is treated as a dielectric continuum.^20^ While continuum‐based models provide a description of the energetics of solvation, the dynamic effects of solvent friction may be lost, leading to an incorrect description of time‐dependent properties. In a Brownian dynamics simulation, these frictional forces are introduced in a stochastic manner that represents the buffeting of solutes by the thermal motion of solvent atoms. Ermak and McCammon^21^ introduced an algorithm to describe the propagation of a system of *N* Brownian particles, in which the displacement of particle *i* during a time step Δt is given by

(1) $$\Delta r_{i}\;=\;\Delta t\sum_{j=1}^{N}\left(\frac{\partial {\overset{⁁}{D}}_{ij}}{\partial r_{j}}\;+\;\frac{{\overset{⁁}{D}}_{ij}}{k_{B}T}\cdot F_{i}\right)+\;R_{i}$$

where *k*_B_ and *T* are the Boltzmann constant and simulation temperature, respectively, ${\overset{⁁}{D}}_{ij}$ is the 3 × 3 hydrodynamic diffusion tensor^22^, ^23^, ^24^ between particles *i* and *j*, $F_{i}$ is the systematic force acting on particle *i*, and $R_{i}$ is a vector describing a random displacement of *i*, sampled at each simulation step from a Gaussian distribution of mean zero that satisfies the variance–covariance relation $〈R_{i}R_{j}〉=2{\overset{⁁}{D}}_{ij}\Delta t$ for all *i* and *j*. The vector $R_{i}$ is obtained by Cholesky decomposition of the *N* × *N* total diffusional tensor $\overset{⁁}{D}$ of the system, whose elements are the pairwise tensors, ${\overset{⁁}{D}}_{ij}$. This decomposition scales as *O*(*N* ^3^), and is the most computationally demanding step of such a Brownian dynamics scheme.

In SDA 7, we model solutes using all‐atom rigid conformations, which allows the use of precomputed interaction grids to speed up the calculation of the systematic forces, $F_{i}$ (See Appendix A). This also removes the need to model intramolecular hydrodynamic interactions, so in dilute solutions, hydrodynamic interactions can be assumed to be negligible, meaning the off‐diagonal tensors in $\overset{⁁}{D}$ can be taken as zero, while diagonal tensors ${\overset{⁁}{D}}_{ij}$ for *i* = *j* can be replaced by scalar quantities. Equation (1) can therefore be simplified to

(2) $$\Delta r_{i}=\frac{\Delta t}{k_{B}T}D_{i}^{T}\cdot F_{i}+R_{i}$$

where $D_{i}^{T}$ is the infinite dilution translational diffusion coefficient of solute *i*, and $R_{i}$ is a random vector of mean zero and variance $〈R_{i}^{2}〉=6D_{i}^{T}\Delta t$. An analogous equation is used to describe the rotation of the rigid solute *i*, in which the translational diffusion coefficient is replaced by the rotational coefficient $D_{i}^{R}$ and the forces are replaced by torques.^6^

Two simulation schemes are used in SDA 7, one that models the bimolecular association of a pair of solutes,^6^ and one which models the dynamics of many solute molecules in solution.^11^ In the first scheme, one of the solutes remains fixed at a position in the centre of the simulated volume, but is allowed to rotate, while the diffusion of the other is simulated. To account for the diffusion of the fixed solute, a combined translational diffusion coefficient is used for the mobile solute, given by the sum of the coefficients of the two solutes. This approach is used to calculate diffusional association rate constants,^6^, ^7^, ^25^ using the Northrup–Allison–McCammon method,^26^ electron transfer rates,^9^ and for docking to obtain the structures of encounter complexes.^8^ In the second scheme, the dynamics of many solutes are modeled, in a simulated volume that may be either periodic or nonperiodic, allowing the effects of solute concentrations to be included. As the total concentration of the solutes increases, the assumption that hydrodynamic interactions can be taken as negligible becomes less valid. To include some of the effects of hydrodynamic interactions, while avoiding the computational expense of the full hydrodynamic diffusion tensor approach described in eq. (1), Mereghetti et al.^12^ introduced a mean‐field approximation to hydrodynamic interactions, which is included in SDA 7. Following a method first described by Heyes,^27^ the diffusion coefficient of solute *i* is scaled at each simulation time step according to the fractional occupancy of the local volume surrounding *i* by other solutes. In calculating the fractional occupancy of local volumes, all solutes are assumed to be spherical, with the radii of their volumes given by their Stokes hydrodynamic radii.

## New Features in SDA 7

### Flexible solute representations

In previous versions of SDA, each solute has been represented by a single rigid conformation. Although a rigid approximation is suitable in many cases, it is known that docking poses can be very sensitive to the initial structures of the interacting solute partners, as solutes may undergo conformational changes during the binding process. Similarly, association rates may be affected by conformational selection or adaptation which is not accounted for in a rigid model.

In SDA 7, solutes can be represented by a set of rigid conformations. If available, these may be a set of structures determined by crystallography or NMR. Alternatively, they may be generated by modeling or simulation. For example, a normal mode analysis may be performed to predict the biologically relevant low frequency motions of a protein, from which a set of representative conformations may be generated. A set of different conformations of a given solute may also be used to represent different protonation states of titratable groups at one or several pH values.

### Potential of mean force calculations

SDA 7 is able to perform potential of mean force (PMF) calculations of the molecular adsorption of solutes to a planar surface. The implementation of PMF is based on a thermodynamic integration method described by Kokh et al.^10^ Given a rigid body structure of a molecule, this method describes the free energy of adsorption as a six dimensional function of energy (three of which define the rotational degrees of freedom and the remaining three the translational degrees of freedom). This configurational space is sampled using a set of rotation angles and positions over an integration interval with predefined subintervals of equal width for each of the six degrees of freedom. As the calculation of a complete six dimensional free energy landscape is usually not feasible, exploration of the configurational space can be restricted to a small area over the surface, if it retains a periodic unit of structure. The reaction coordinate in the PMF calculations is defined as a line perpendicular to the surface.

### Updated preparation and analysis tools

The set of tools available in SDA 7 has also been extended and unified for both bimolecular and many‐molecule simulations. These include preparation tools for creating the 3‐dimensional interaction potential grids, for calculating effective charges^28^ used to represent the solutes, and for generating initial configurations with many solutes at a given concentration. The analysis tools include tools for postanalysis of trajectories, for example to calculate translational and rotational self‐diffusion coefficients, and conversion tools to convert SDA 7 trajectory files and structure files to PDB or DCD formats, or between UHBD and DX grid file formats.

## Details of the SDA 7 Implementation

SDA 7 has been rewritten in Fortran 90 (F90) to allow the use of language features that were not available in the previous Fortran 77 (F77) versions of SDA. Dynamic memory allocation allows for more efficient memory use, and for larger systems to be simulated, limited only by the available system memory. F90 modules provide a modular framework to the code, allowing library functions to be reused in various parts of the main simulation code and that of the related preparation and analysis tools. The use of modules and pointers also allows related data structures to be grouped together producing an object‐oriented structure. Unlike in previous versions of SDA, bimolecular simulations may now be run in parallel, through the use of OpenMP.

### Solute representation

**Figure 1:**
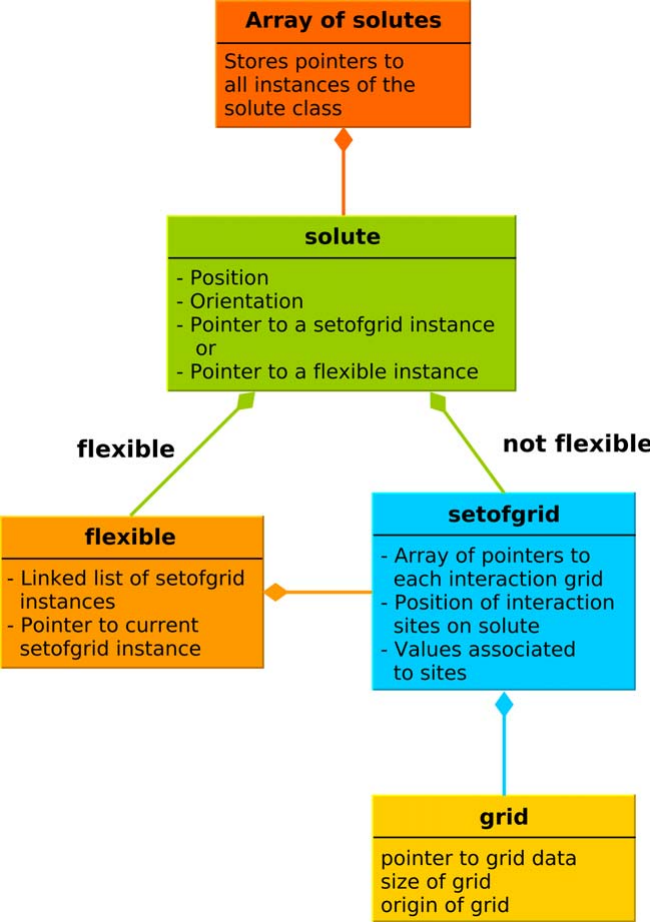
Simplified class diagram of the solute‐grid relationship (and the memory storage of simulation data). An array contains pointers to each instance of the *solute* class for each solute. A single *setofgrid* instance is used for all identical conformations of the solutes to minimize memory requirements. In the case of flexible solutes, an intermediate *flexible* object containing a linked‐list of pointers to the *setofgrid* instances of each conformation is added to each flexible solute.

Figure 1 shows the relationships between the module classes used to represent a set of solutes. During SDA 7 simulations, the precomputed interaction grids constitute the largest memory requirement of the calculation. In the case of a simulated system containing many identical solute molecules, it is desirable to store only one copy of their identical grids. Furthermore, when a solute is represented by more than one rigid conformation, the grids corresponding to the different conformations of the same solute should be grouped together in a single class.

One conformation of a solute is described by the *setofgrid* class. It comprises the 3D precomputed interaction potential grids (yellow in the figure) as well as the positions and values of their interaction sites, such as effective charges or atomic surface accessibilities, within the 3D solute structure. Additional information is also stored here, such as reaction criteria to define encounter complexes, the solute infinite dilution diffusion coefficients used for BD propagation or the Stokes hydrodynamic radii used in the mean‐field hydrodynamic approximation. Each *solute* object only needs to store the instantaneous position and orientation of the solute and a pointer to its associated *setofgrid* instance. When a solute is defined by more than one conformation, an intermediate *flexible* object (shown in orange) is associated to each flexible solute. It consists of a linked list of *setofgrid* instances, each corresponding to a specific conformation. Upon a change of conformation, the pointer to the current conformation is updated to the correct *setofgrid* instance. Finally, pointers to all solute instances are stored in an array (shown in red). This design provides a simple and memory efficient way to simulate many different solute types, each of which may be represented by a set of conformations.

### Algorithm for treating solute flexibility

When simulating solutes defined by multiple coordinate sets, these coordinates are stored in a linked list. After the update of the solutes' positions and orientations during the BD propagation step, trial moves to alternative coordinate sets may be attempted. The time interval between trial moves can be set to either be fixed period, or can be drawn from a normal distribution, with the mean and standard deviation of this distribution set by the user in the input file.

A parameter defined in the input file, *nearest*, can limit a trial move to an adjacent conformation in the list, or specify that trial moves may be attempted to a randomly chosen conformation. The use of adjacent conformations may be preferable when the conformations are generated by normal mode analysis, but may introduce a bias in the sampling of the first and last conformation in the list. When the conformations are obtained from NMR or crystal structures, a random change of conformation may be preferable.

SDA 7 includes three acceptance criteria for deciding whether a trial move should be accepted: (i) to always accept the move, (ii) to accept all moves that lower the interaction energy of the solute, and (iii) a Metropolis algorithm^29^ in which conformational changes that lower the interaction energy of that solute are always accepted, while those that increase the interaction energy are accepted with a probability $\text{exp}(\frac{\Delta G_{0}-\Delta G_{1}}{k_{B}T})$, where $\Delta G_{0}$ is the interaction energy of the solute with its surroundings in the old conformation and $\Delta G_{1}$ is the interaction energy of the new conformation.

### SDA 7 workflow and parallelization

**Figure 2:**
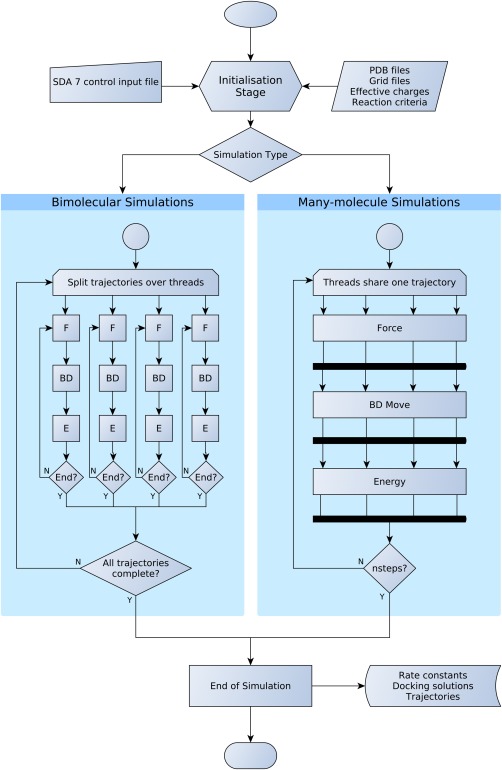
Workflow of an SDA 7 simulation. The simulation parameters are set in a single control input file, which also defines the location of the additional files needed for the simulation. The simulation proceeds through one of two routes, depending on whether it is a bimolecular or many‐molecule simulation. With bimolecular simulations looping over many trajectories with different initial configurations, and many‐molecule simulations consisting of a single trajectory of *nsteps* BD moves. In both simulation types, there are three main calculations that may be computed within a simulation time step: a force calculation (F), a BD propagation move (BD), and an energy evaluation (E). The simulation types are parallelized differently (shown here as an example with four OpenMP threads), with each thread in a bimolecular simulation running an independent trajectory and the force, BD and Energy calculations parallelized across threads in many‐molecule simulations. In many‐molecule simulations, all threads must synchronize between these three calculations. Both types of simulation produce output files that are consistent with each other. [Color figure can be viewed in the online issue, which is available at wileyonlinelibrary.com.]

The workflow of an SDA 7 simulation is shown in Figure 2. The initialisation stage is common to all simulation types, with simulation parameters defined in a single control input file. This file also defines the location of any additional files required for the calculation, such as structure files and files describing the precomputed solute interactions.

The main calculation loop is processed differently for bimolecular and many‐molecule simulations, mainly because a different OpenMP parallelization scheme is required for these different simulation types. During bimolecular simulations, SDA can compute association or electron transfer rate constants and predict the structures of the most energetically favorable docked encounter complexes. During many‐molecule simulations, the radial distribution functions can be computed on the fly, while other properties, like translational and rotational self‐diffusion coefficients or transient oligomerizations, are computed with separate tools in a postprocessing stage.

SDA 7 has been developed to exploit multicore shared‐memory architectures with the use of OpenMP. During bimolecular simulations, thousands of trajectories are typically generated to accumulate the required statistical data. In this case, a simple parallelization scheme is possible, with each thread computing a single trajectory at a time. Each thread needs to have its own private copy of the solutes, with independent positions and orientations, but they can share the same grid data, reducing the overall memory cost compared to either running the trajectories as independent jobs, or using a Memory Passing Interface parallelization scheme. There are some scaling bottlenecks due to accessing shared arrays during the simulations. For example, during docking simulations, each thread owns a private list of high scoring complexes that have been computed. At regular intervals, these lists are merged into a shared one. An additional factor that affects scaling is what we call the “end‐effect.” At the end of a bimolecular simulation, only one thread is executing a trajectory, the others have no remaining trajectories to run, and remain idle. In practice, the end‐effect is usually small, as shown in the benchmarks described in the results section.

In many‐molecule simulations, single trajectories are generated. The calculation of the forces on each solute represents the most computationally expensive step (typically more than 95% of the CPU time). The algorithm, which formally scales as $O(N^{2})$ (or *O*(*N*) with the use of cutoffs) with *N* being the number of solutes, is a double loop over pairs of solutes. OpenMP is used for distributing the first loop across the number of available cores. The calculation of energies and the trajectory propagation is parallelized similarly.

Simulations can be performed within a spherical volume or a rectangular box, with or without periodic boundary conditions. An atomically detailed planar surface, lying in the *xy*‐plane may be defined in simulations.

## Application examples and benchmarks

**Figure 3:**
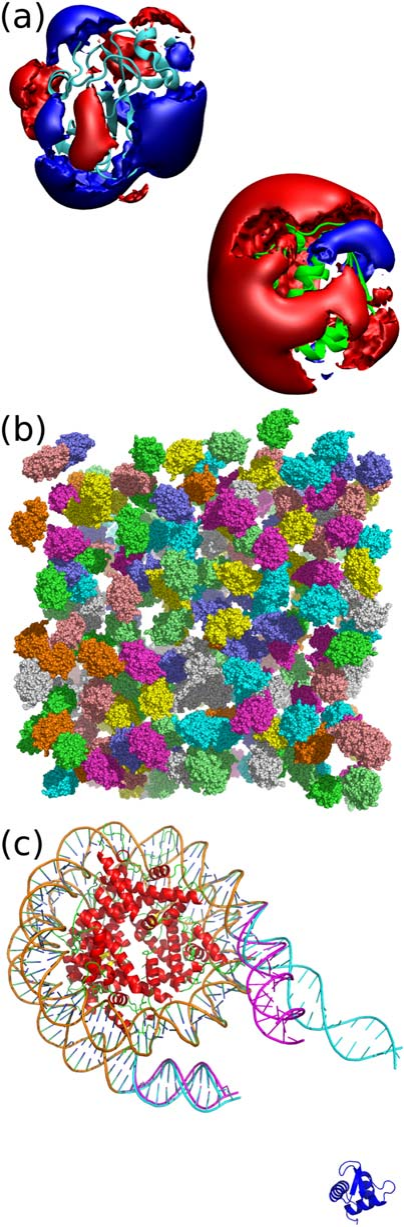
Test systems used for the validation and benchmarking of SDA 7. a) The electrostatically guided bimolecular association of barnase (cyan) and barstar (green). The + 0.5 kcal/(mol ·e) (blue) and –0.5 kcal/(mol ·e) (red) isosurfaces of the electrostatic potentials surrounding each solute are shown. b) A snapshot of the simulation of 256 HEWL molecules in solution (The HEWL molecules are colored differently for clarity). c) Docking of the globular domain of the linker histone H5 (blue) to the nucleosome. The crystal conformation (magenta, 7_0_) and a structure generated with an elastic network model (cyan, 7_6_) of the flexible region of the nucleosome are shown.

In this section, we discuss simulations of three systems that highlight different functionalities available in SDA 7 (Fig. 3). First, we consider the association of the two proteins, barnase and barstar (Fig. 3a). Barnase is an extracellular ribonuclease and barstar is its intracellular inhibitor. They are known to have a very strong binding affinity, with a fast bimolecular association rate constant of 10^7^−10^9^ M^−1^s^−1^,^30^, ^31^ depending on experimental conditions. Barnase and barstar already served as a model protein pair in the development of SDA^6^, ^7^, ^8^, ^25^ because the wild‐type proteins and a number of mutants have been extensively characterized biochemically and structurally. We use the barnase–barstar system to investigate the parallel scaling of SDA 7, during both docking and association rate calculations. We also compare the single thread speed with that obtained using SDA 6.

The second system is a set of Hen Egg White Lysozyme (HEWL) protein molecules whose diffusion is simulated using the many‐molecule simulation functionality of SDA 7 (Fig. 3b). Mereghetti et al.^11^ previously reported the use of SDAMM to study the dynamics of HEWL in solution at different protein concentrations. They compared osmotic second virial coefficient *B*_22_ values at three different pH values (3, 6, and 9) and at different ionic strengths, and the self‐diffusional coefficients at pH 4.6 and at varying protein concentrations. This system was also used to validate the implementation of the Debye‐Hückel electrostatic correction used in both SDAMM and SDA 7.^32^ We use this example system to assess the scaling of many‐molecule simulations in SDA 7, and examine the effect that including a flexible solute representation, by allowing the HEWL molecules to switch between their protonation states at pH 3, 6, and 9, have on the scaling of the method. While modeling transitions between pH‐dependent charge states is somewhat artificial, we use the results of these simulations to discuss the potential future applications of this novel method.

Finally, we consider the docking of the globular domain of the linker histone H5 to a flexible nucleosome (Fig. 3c), which was previously studied by Pachov et al.^33^ This application case shows the use of SDA 7 for a large system, with one solute treated flexibly. Pachov et al.^33^ generated a series of conformations from a normal mode analysis using an elastic network model of the nucleosome, and independent BD simulations were performed for each conformation. With the flexibility module of SDA 7, these simulations can be performed in a more efficient way. Treating solute flexibility in a single simulation allows for only those complexes with the most favorable binding energies to be recorded, whichever conformation of the flexible solute they include. Furthermore, with a suitable conformational sampling algorithm, sampling can be biased towards those conformations that produce encounter complexes with more favourable binding free energies, reducing the total simulation time required.

### Methods

SDA 7 was compiled using gfortran 4.8.1. The barnase–barstar and HEWL benchmark simulations were run on a compute cluster consisting of nodes containing four AMD Opteron 6174 12‐core 2.2 GHz processors. For comparison, SDA 6 was compiled with both gfortran 4.8.1 and ifort 11.1, and simulations were performed on the same compute cluster. The docking simulation of the linker histone H5 to the nucleosome was performed on a cluster node containing two Intel Xeon E5‐2630 6‐core 2.3 GHz processors.

#### Case I: Bimolecular simulation of barnase–barstar association

The coordinates of barnase and barstar were taken from a crystal structure of their complex (PDB code: 1BRS, 2.0 Å resolution). Protonation states for each protein were assigned at pH 7 using PDB2PQR^34^, ^35^ resulting in net charges of + 2 *e* for barstar and –6 *e* for barnase. Atomic partial charges and radii were taken from the Amber force field.^36^ Electrostatic potential grids of size 129^3^ Å^3^ and spacing 1 Å were generated with APBS^37^ by solution of the linearized Poisson–Boltzmann equation with an ionic strength of 50 mM, ion radius of 1.5 Å, a protein interior dielectric constant of 4.0, a solvent dielectric constant of 78.0 and a temperature of 298.15 K. Effective charges^28^ on each protein were calculated using the *ECM* module, and electrostatic and hydrophobic desolvation grids of size 110^3^ Å^3^ and spacing 1 Å were created with the *make_edhdlj_grid* module of SDA 7.

Bimolecular association rate and docking simulations were performed using SDA 7 with processor core counts ranging from 1 to 48. For each simulation type and core count, the simulations were repeated 10 times using different random number generator seeds. In each simulation, 20,000 independent trajectories were created. Ten simulations of 20000 trajectories were also performed with SDA 6 (compiled with both gfortran and ifort), and compared to the single processor simulations using SDA 7. In all simulations, the initial position and orientation of barstar was randomly chosen to lie on a sphere of radius 150 Å, centred on the geometric midpoint of barnase, and all simulations were terminated when the interprotein centre‐to‐centre separation reached 300 Å.

During the simulations for the calculation of the bimolecular association rate constant, long‐range electrostatic and short‐range electrostatic desolvation interactions (see Appendix A) were acting between the proteins. The association rate constant *k*_on_ was calculated using the Northrup‐Allison‐McCammon method,^26^ with an encounter complex defined when two independent native contacts were sampled at a distance of 6 Å, as used previously by Gabdoulline and Wade.^25^ Pairs of native contacts were considered as being independent if the interacting residues of the same protein were separated by more than 5 Å.

To better describe the binding energetics of close protein contacts, in addition to the long‐range electrostatic and short‐range electrostatic desolvation interactions used in the rate calculations, short‐range hydrophobic desolvation interactions were also modeled during the docking simulations, during both the BD search and in scoring the docked complexes. Five thousand low energy docked encounter complex structures were recorded during the simulation, and subsequently clustered using an average‐linkage hierarchical clustering method developed by Motiejunas et al.^8^ Encounter complex solutions that lay within 1 Å RMSD of a previously recorded lower energy solution were not recorded, but were counted to allow the sampling of cluster populations to be determined correctly. Cluster representatives were compared to the crystallographic structure of the complex (PDB code 1BRS), by aligning the backbone atoms in barnase and calculating the RMSD of barstar backbone atoms. Some docked cluster representatives were further refined by low temperature MD using the Amber ff99SB force field of Hornak et al.^38^ and the GBNeck implicit solvent model of Mongan et al.^39^ The structures of the docked encounter complexes were first energy minimized, before MD refinement. Initial velocities were drawn from a Maxwell‐Boltzmann distribution at 30 K, then heated to 200 K over 50 ps, simulated at 200 K for a further 50 ps, then cooled back to 30 K over 200 ps. The final complex structures obtained were then compared to the known native docked complex (PDB: 1BRS) and their binding affinities estimated using the same GB model used during the simulations.

#### Case II: Many‐molecule simulations of hen egg white lysozyme

A crystal structure of HEWL (PDB code: 1HEL, 1.7 Å resolution) was used to assess the performance of many‐molecule simulations in SDA 7. The protonation states of HEWL at pH 3, 6, and 9 were assigned from experimental p*K*_a_ values, as described by Mereghetti et al.,^11^ giving net charges of + 13 *e*, + 8 *e*, and + 7 *e*, respectively. Atomic partial charges and radii were assigned from the PARSE force field.^40^ Long‐range electrostatic potential grids of size 97^3^ Å^3^ with a 1 Å resolution were created by solving the linearized Poisson–Boltzmann equation with APBS,^37^ using an ionic strength of 5 mM, an ion radius of 1.5 Å, a protein dielectric constant of 2.0, a solvent dielectric of 78.0 and a temperature of 298.15 K. Effective charges were calculated using the *ECM* module of SDA 7, while short‐range electrostatic and hydrophobic desolvation, and soft‐core repulsive grids, of size 97^3^ Å^3^ and 1 Å spacing, were created using the *make_edhdlj_grid* module. An initial configuration of 256 HEWL molecules was created using the *genbox* tool in SDA 7 at a protein concentration of 150 mg/ml.

Two sets of simulations were performed to investigate the scaling performance of the SDA 7 code. In the first, all 256 HEWL molecules were simulated in their pH 6 protonation state. In the second, each HEWL molecule was randomly assigned to a pH 3, 6, or 9 protonation state at the beginning of the simulations, and transitions between states were performed at regular intervals during the simulations. Two algorithms for changing states were used, one where trial changes in the state of a protein were accepted if they resulted in a lowering of the interaction energy of that protein with its surroundings, and another where trial moves were accepted according to the Metropolis criterion.^29^ All simulations were performed using processor core counts ranging from 1 to 48, with each simulation repeated 10 times with different initial random seeds. All simulations were performed for 10 ns using a 0.25 ps time step, with hydrodynamic interactions accounted for using a mean‐field approach^12^ and longer range electrostatic interactions modeled using a Debye–Hückel correction term.^32^

Two longer 500 ns BD simulations were also performed during which each HEWL molecule was intially assigned to a randomly chosen charge state. These states were allowed to change during the simulations according to either the minimum energy or Metropolis criteria. The fractional occupancies of each charge state were then examined during the simulation trajectories.

#### Case III: Docking of the linker histone H5 to the nucleosome

The initial conformation of the nucleosome was generated from a crystallographic structure of the core nucleosome particle (PDB code: 1KX5, 1.9 Å resolution), with the linkers extended by 20 base pairs using a lower resolution structure (PDB code: 1ZBB, 9 Å resolution). Normal‐mode analysis with an elastic network model was performed using the Nomad‐Ref webserver,^41^ to generate a flexible representation of the linker regions, as described by Pachov et al.^33^ Seven structures were selected to represent the lowest frequency vibrational mode (7_0_ to 7_6_ in Ref^33^). The structure of the globular domain of the linker histone H5 (gH5) was taken from the crystal structure (chain B, PDB code: 1HST, 2.5 Å resolution).

Only long‐range electrostatic interactions were taken into account in the simulation. Titratable residues were protonated using PDB2PQR^34^, ^35^ with the Amber force field,^36^ resulting in net charges of –237 *e* for the nucleosome and + 11 *e* for gH5. The electrostatic potential grids were calculated with APBS,^37^ by solving the nonlinearized Poisson–Boltzmann equation at an ionic strength of 0.1 M, with an ion radius of 1.5 Å, a solute dielectric constant of 2.0, a solvent dielectric of 78.54 and a temperature of 298.15 K. Grid sizes were 257^3^ Å^3^ for the nucleosome and 129^3^ Å^3^ for gH5, with a 1.0 Å resolution. Effective charges were calculated using *ECM*.

The docking simulation consisted of 25,000 trajectories with a maximum simulation time of 100 ns for each trajectory. The initial centre‐to‐centre distance between the nucleosome and gH5 was set to 280 Å and the trajectories were stopped when the distance between the centres of the solutes reached 500 Å. Docked complexes were recorded when the centre‐to‐centre distance was within 73 Å and the centre of gH5 was within 40 Å of the nucleosome dyad point, as defined by Pachov et al.^33^ Trial moves to adjacent nucleosome conformations, along the lowest frequency normal mode, were attempted at a fixed interval of 1.0 ns, and accepted according to the Metropolis algorithm.

The 20,000 lowest energy docked complexes were recorded for subsequent clustering. Complexes obtained using each nucleosome conformation were clustered separately using the clustering algorithm described by Motiejunas et al.^8^ Docked structures were only recorded if they had a RMSD larger than 1 Å, compared to a previously recorded lower energy solution, but all complexes satisfying the above distance criteria were counted to provide correct cluster populations.

### Results

#### Case I: Bimolecular simulation of barnase–barstar association

**Figure 4:**
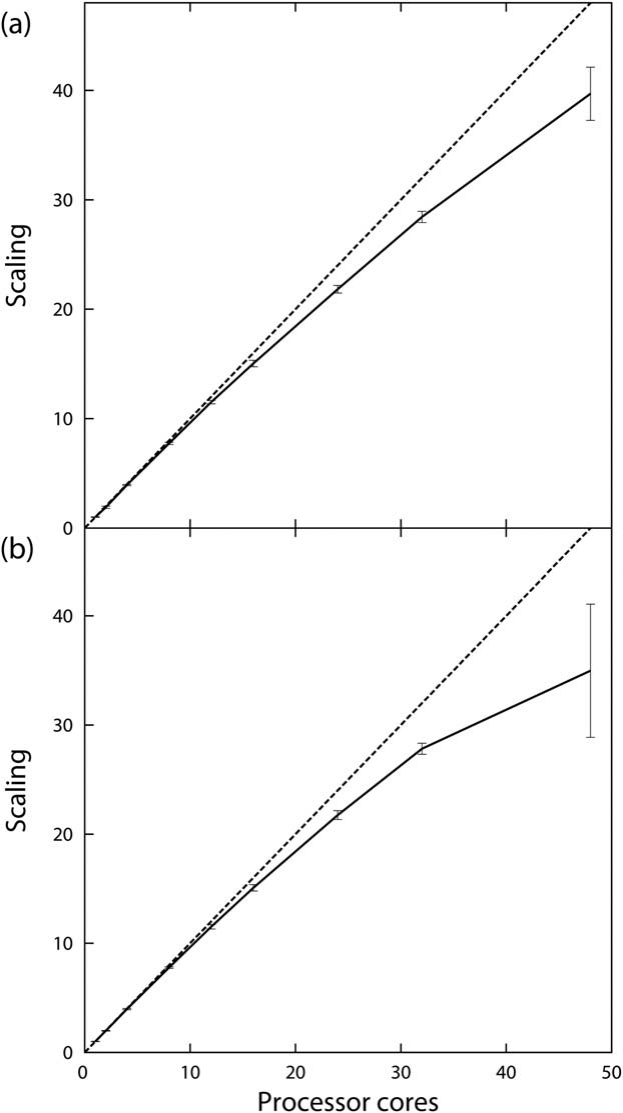
Scaling performance for simulations of barnase and barstar to compute a) the bimolecular association rate constant and b) the structures of docked complexes. Error bars show standard deviations across 10 independent simulations performed for each processor count. Dotted lines indicate perfect scaling.

Calculations of the bimolecular association rate constant were performed for barnase and barstar (Table 1, Fig. 4). The association rate constants calculated ranged from 2.7 to $2.9\;\times 10^{8}$ M^−1^s^−1^ (Table 1), in good agreement with experimental values, and values computed with previous versions of SDA. The SDA 7 code scales well up to 48 cores, as used in the benchmark, with a scaling factor of 39.7 and a scaling efficiency of 83%. The single core performance is significantly improved in SDA 7 with an average execution time of about 5000 s, compared to approximately 10,000 s and 80,00 s, for SDA 6 compiled with gfortran 4.8.1 and ifort 11.1, respectively.

**Table 1:** Scaling of the Case I (barnase–barstar) benchmarks on different numbers of cores.

Number of cores	Association			Docking		
	Time^a^	Scaling^b^	*k*_on_ c	Time^a^	Scaling^b^	RMSD^d^
1	5128 ± 126	1.00 ± 0.02	2.7 ± 0.2	31461 ± 503	1.00 ± 0.02	4.9 ± 0.4
2	2705 ± 154	1.90 ± 0.11	2.7 ± 0.1	15886 ± 250	1.98 ± 0.03	4.9 ± 0.4
4	1307 ± 16	3.92 ± 0.05	2.8 ± 0.3	7933 ± 115	3.97 ± 0.06	4.8 ± 0.4
8	663 ± 9	7.73 ± 0.10	2.7 ± 0.2	4047 ± 36	7.77 ± 0.07	4.9 ± 0.4
12	446 ± 6	11.50 ± 0.16	2.8 ± 0.3	2732 ± 48	11.51 ± 0.21	4.9 ± 0.4
16	341 ± 7	15.04 ± 0.30	2.8 ± 0.3	2085 ± 40	15.09 ± 0.29	4.8 ± 0.4
24	235 ± 4	21.83 ± 0.35	2.8 ± 0.2	1446 ± 27	21.75 ± 0.41	4.9 ± 0.4
32	180 ± 3	28.43 ± 0.52	2.8 ± 0.2	1130 ± 20	27.82 ± 0.51	4.8 ± 0.2
48	129 ± 8	39.69 ± 2.43	2.9 ± 0.3	899 ± 157	34.97 ± 6.10	4.9 ± 0.3

a Mean simulation wall‐time (s) with standard deviation.

b Mean simulation performance scaling, relative to mean single core time, with standard deviation.

c *k*_on_ (10^8^ M^−1^s^−1^) calculated with an encounter complex definition of 2 independent native contacts within 6 Å, (see methods).

d Root mean squared deviation (Å) of representative of second most populated cluster of barstar to barstar in the complex in the PDB file, 1BRS.

In separate simulations, the diffusional encounter complex structure was predicted, using SDA 7 to dock barstar to barnase (Table 1). The 1000 most energetically favorable complexes, with atomic RMSDs of greater than 1 Å from each other, from the 20,000 trajectories of each simulation were clustered into five clusters. The second most populated cluster, with a population of approximately 40% of complexes sampled during the simulation, agreed closely with the crystal structure, with a backbone RMSD of the cluster representative of barstar of 4.8–4.9 Å. This cluster was also ranked second energetically. In the most populated cluster, the barnase‐barstar interface was rotated approximately 90 °, relative to the crystal structure. The representative clusters from the second most populated cluster of each of the 90 SDA 7 simulations (simulations with nine different numbers of processor cores, repeated 10 times each) were relaxed with low temperature MD. The RMSD of the interfacial residues of barstar, relative to barnase, was monitored during the simulations by considering all barstar heavy atoms within 6 Å of barnase in the crystal structure. Prior to simulation, the RMSD of these atoms in the 90 cluster representatives, relative to the crystal structure, was between 3.8 and 4.3 Å. While some complexes relaxed to structures further from the native complex, 71 relaxed to conformations in which the RMSD of barstar's interfacial heavy atoms was below 3.5 Å, relative to the crystal structure, with the lowest having an RMSD of 1.9 Å. The structure with the most favorable binding affinity, as calculated from the final complex structure using the same GB parameters as were used in the simulation, had an interfacial heavy atom RMSD of 2.6 Å.

During this docking benchmark, SDA 7 scales up to 48 processor cores with a scaling factor relative to one core of 35.0, a 73% scaling efficiency. The single core performance is somewhat improved in SDA 7 with an average execution time of about 31,000 s, compared to approximately 40,000 s and 32,000 s, for SDA 6 compiled with gfortran 4.8.1 and ifort 11.1, respectively.

#### Case II: Many‐molecule simulations of hen egg white lysozyme

**Figure 5:**
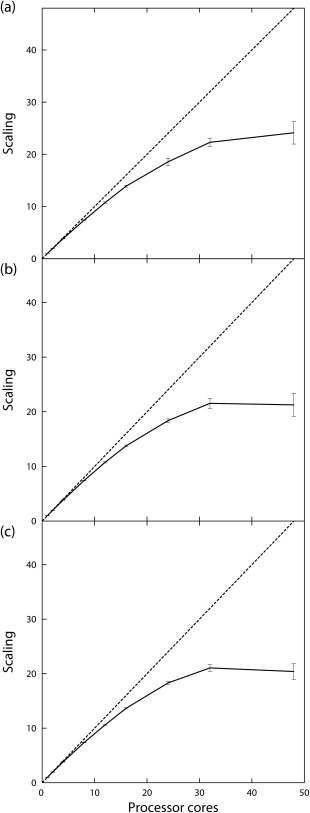
Scaling performance for 10 ns simulations of 256 HEWL molecules a) with residue protonation states calculated at pH 6, and with protonation states at pH 3, 6 and 9 with conformational switching using the b) minimum energy, and c) Metropolis algorithms. Error bars show standard deviations across ten independent simulations performed for each processor count. Dotted lines indicate perfect scaling.

Simulations of 256 HEWL molecules were performed first with protonation states fixed to those assigned at pH 6, and then with each molecule able to switch between protonation states assigned at pH 3, 6, and 9, at intervals during the simulation. The simulation wall time for processor core counts ranging from 1 to 48 was obtained and the scaling factor calculated from the mean time required to run the simulation on one core (Table 2, Fig. 5). For each of the three simulation types, we see reasonable scaling up to 32 cores, but little or no improvement in performance when increasing the core count to 48. The simulation times on a given number of processors were very similar for the three simulation types.

**Table 2:** Scaling of the Case II (HEWL) benchmarks on different numbers of cores.

Number of cores	Single protonation state		Three protonation states			
			Minimum energy algorithm		Metropolis algorithm	
	Time^a^	Scaling^b^	Time^a^	Scaling^b^	Time^a^	Scaling^b^
1	6426 ± 83	1.00 ± 0.01	6480 ± 217	1.00 ± 0.03	6431 ± 126	1.00 ± 0.02
2	3273 ± 30	1.96 ± 0.02	3337 ± 53	1.94 ± 0.03	3300 ± 44	1.95 ± 0.03
4	1673 ± 18	3.84 ± 0.04	1695 ± 18	3.82 ± 0.04	1675 ± 15	3.84 ± 0.03
8	872 ± 8	7.37 ± 0.07	878 ± 3	7.38 ± 0.03	866 ± 7	7.43 ± 0.06
12	599 ± 9	10.74 ± 0.16	611 ± 5	10.61 ± 0.09	598 ± 7	10.75 ± 0.12
16	463 ± 6	13.89 ± 0.18	475 ± 4	13.65 ± 0.12	468 ± 6	13.76 ± 0.17
24	346 ± 12	18.56 ± 0.67	354 ± 5	18.31 ± 0.25	350 ± 6	18.38 ± 0.32
32	288 ± 10	22.30 ± 0.78	308 ± 9	21.06 ± 0.62	298 ± 13	21.55 ± 0.93
48	266 ± 24	24.13 ± 2.16	318 ± 22	20.40 ± 1.43	302 ± 30	21.26 ± 2.11

a Mean simulation wall‐time (s) with standard deviation.

b Mean simulation performance scaling relative to mean single core time with standard deviation.

During the longer 500 ns simulations, the HEWL charge states rapidly converged to the pH 9 charge states, that is, the state in which HEWL has the lowest overall charge. During the simulation in which trial transitions were accepted according to the minimum energy criterion, after 10 ns all HEWL molecules were in this state, and remained so for the rest of the simulation (Supporting Information Fig. S1). During the simulations that used the Metropolis acceptance criterion, the charge states converged more slowly. After approximately 75 ns, all HEWL molecules were in the pH 9 states, with only occasional sampling of the other two states in the latter parts of the simulation (Supporting Information Fig. S2).

#### Case III: Docking of linker histone H5 to the nucleosome

The docking of gH5 to the nucleosome took approximately 3.5 h to complete on 12 processor cores. For each nucleosome conformation, the docked complexes were clustered into 10 clusters. Table 3 shows the number of docked complexes obtained for each nucleosome conformation, the population of the first docked cluster, according to the total simulation sampling, and the backbone RMSD of gH5 in the representative of the first cluster and in the docked representative structure obtained by Pachov et al.^33^

**Table 3:** Clustering of Case III (linker histone H5‐nucleosome) docking.

Conformation	Number of solutions	First cluster population (%)	RMSD^a^
7_0_	248,678	82	3.4
7_1_	176,171	84	5.4
7_2_	43,138	85	8.0
7_3_	45,952	83	7.5
7_4_	22,960	37	7.6
7_5_	112,010	71	5.5
7_6_	90,214	59	19.5

a Backbone RMSD (Å) of gH5 in the representative of the first cluster and the structure obtained by Pachov et al.

The majority of the high scoring complexes were obtained with nucleosome conformations 7_0_ (corresponding to the crystal structure) and 7_1_. In both cases, clustering the complex docking solutions obtained with these nucleosome conformations resulted in the most populated clusters containing approximately 80% of the sampled complexes. The lower number of docked complexes for the other conformations of the nucleosome suggests they formed weaker interactions. The representative of the first cluster obtained with conformation 7_0_ is in reasonable agreement with the reference structure published by Pachov et al.,^33^ showing a backbone RMSD of 3.4 Å. An exact agreement cannot be expected due to differences in the clustering procedures and the number of trajectories sampled. Other conformations show representative structures somewhat shifted from the reference position, with the last 7_6_ conformation giving a first docked cluster far away from the reference binding site. Pachov et al. found a similar distribution of RMSD values over the conformations of this nucleosome mode with a very different binding location for the linker histone to conformation 7_6_ compared to all the other conformations for this mode (see Fig. 2a in Pachov et al.^33^)

### Discussion

SDA 7 has been completely rewritten in order the combine the existing SDA and SDAMM codes into a single framework. A new modular and object‐oriented approach has been used to allow for easier integration of new methods. The new code uses dynamic memory allocation and improved parallelization to allow increasingly large systems to be investigated, up to the limits imposed by the operating environment.

The results of the application cases presented are consistent with previously published work, both computational and experimental, and show no variation with respect to the number of processor cores used for the calculations. The bimolecular association rate constant for the diffusional association of barnase and barstar was calculated to be 2.7– $2.9\times 10^{8}$ M^−1^s^−1^, in close agreement with the experimentally derived value of $2.86\times 10^{8}$ M^−1^s^−1^, obtained at pH 8 and 50 mM NaCl.^31^ This value compares favorably to the $3.88\times 10^{8}$ M^−1^s^−1^ reported by Gabdoulline and Wade in a study that used a previous version of SDA to model identical experimental conditions.^25^ The difference is likely due to sampling differences and the use of different force fields for the initial atomic partial charge and radius assignment, as the underlying method remains the same.

Brownian dynamics docking using SDA 7 was able to predict the native structure of the barnase‐barstar complex to within 4.8–4.9 Å of the known crystal structure, within the second most populated cluster (Table 1). This is consistent with the results of Motiejunas et al.^8^ who previously used SDA to dock barstar to barnase and also found that the native structure agreed more closely with their second most populated cluster. It should be noted that SDA 7 is designed to predict diffusional encounter complexes rather than fully docked complexes. The exclusion grids used in SDA 7 to prevent two molecules from overlapping each other often result in predicted complexes in which the two interacting solutes have slightly increased separations. To assess the docked solutions obtained with SDA, short low‐temperature MD simulations were performed to allow the docked complexes to relax to more stable positions. Following these simulations, the lowest RMSD of barstar's interfacial heavy atoms dropped from 3.8 to 4.3 Å, relative to the native crystallographic structure, in the initial conformations to 1.9 Å, in the lowest case, and 2.6 Å, in the lowest energy case, as estimated from single‐point GB calculations.

The bimolecular BD simulations described here are trivially parallelizable as the large number of trajectories performed can be split between threads easily. This is reflected in the scaling shown in the benchmark simulations. There are, however, some factors that limit scaling. As the simulations are of indeterminate length, it is possible that a long lasting trajectory can result in a single processor core continuing to complete a trajectory while the others remain idle, having completed all other required trajectories. Also, in the case of docking simulations, we keep a global list of the highest scoring docked complexes sampled across threads. Synchronisation of this list requires occasional waiting times for some threads. The effects of this are shown in the barnase‐barstar simulations reported here, where the 48 core docking benchmark shows only a 35‐fold reduction in execution time, compared to a single core simulation, while the association benchmark shows a 40‐fold reduction. Moreover, the new parallelization of SDA 7 does not adversely affect single core performance, indeed, both association and docking simulations in fact complete faster than equivalent simulations with SDA 6.

The parallelization of many‐molecule simulations is more complex, with the force, trajectory propagation, and energy calculation routines requiring that the previous routine has completed for all threads before execution. Each of the 256 HEWL molecule benchmarks reported here show that we obtain reasonable parallel scaling up to approximately 24 cores (Table 2, Fig. 5), with the three benchmarks showing 18‐ to 19‐fold reductions in execution time. In comparison, the 48 core benchmark showed only a 20‐ to 24‐fold reduction. The change of conformation algorithms had no significant effect on scaling, with the scaling penalty appearing to be slightly smaller for the Metropolis algorithm compared to the minimum energy algorithm.

It was expected that the scaling of many‐molecule simulations would be dependent on both the number of proteins within the simulation box and the density of the box. To test this, we ran simulations of 256, 2500, and 5000 HEWL molecules at pH 6 and a concentration of 15.0 mg/ml, on 1, 24, and 48 cores. The simulations were otherwise performed as described previously. For 256 HEWL molecules, we observed 12‐fold and 10‐fold reductions in execution time for the 24 and 48 core runs respectively, relative to the single core run. Comparing to Table 2, this showed that decreasing the protein density, which results in a decrease in the number of force calculations, the most computationally expensive step, resulted in a lower scaling performance. However, for 2500 HEWL molecules at 15.0 mg/ml, we observed 17‐fold and 34‐fold, and for 5000 HEWL molecules, 21‐fold and 38‐fold reductions. Thus, increasing the total number of molecules improved the scaling performance.

During the longer 500 ns simulations, in which each of the 256 HEWL molecules was able to switch between any of the three charge states used in this work, the pH 9 charge state was shown to be overwhelmingly more favorable. This is not surprising, as all HEWL molecules will repel each other due to their overall positive charge, while the pH 9 state has the lowest overall charge ( + 7 *e*, compared to + 8 *e* and + 13 *e* for pH 6 and pH 3, respectively). While this result does not represent a physically realistic experiment, as the different charge states relate to extremes in experimental conditions, we show it as a demonstration of how the ability to model “flexible solutes” can be used. More realistic results could be obtained by allowing transitions between a set of protonation states accessible at a given pH, or between different conformations of a flexible solute, as shown in the docking simulations of the linker histone H5 to the nucleosome. In these simulations, we were able to reproduce previously reported predictions^33^ in a more efficient manner. By allowing the nucleosome to sample a range of conformations during a single simulation, the required simulation time can be reduced, as the simulation is biased towards the most important complex‐forming configurations, and we can obtain the relative sampling of conformations within the lowest energy docked complexes (Table 3).

## Concluding Remarks

SDA 7 is a major update of the SDA software. It has new functionalities including the possibility to account for different conformations of the solutes and parallelization on multi‐core processors for bimolecular simulations. All development branches of SDA have been consolidated into a single, common framework using an object‐oriented, easily extendable approach. The results obtained in the validation cases are consistent with the results of previous published studies.

The SDA 7 code is available for download at http://mcm.h‐its.org/sda7, and is free of charge for noncommercial use. The website includes documentation to guide new users, as well as descriptions of example simulations that are included within the distribution. An SDA 7 webserver (webSDA) is also available at http://mcm.h‐its.org/webSDA.

## Supporting information

Supporting Information

## Appendix A Solute Interaction Force Field in SDA 7

This appendix describes the different force field options available in SDA 7. All terms in the force field are optional and can be chosen in the input file. In general, all force and energy terms are precomputed, and stored in memory in 3‐dimensional grids, or in 1‐dimensional arrays, in the case of isotropic distance‐dependent interactions. An exception to this is a short‐range analytical correction to the method of images charge model used to describe the electrostatic interaction of solutes with conducting surfaces.

In SDA 7, the total interaction energy $\Delta G^{1-2}$ between a pair of solutes, 1 and 2, is represented as

(A1) $$\begin{array}{l} \Delta G^{1-2}=\Delta G_{\text{el}}^{1-2}+G_{\text{edesolv}}^{1-2}+\Delta G_{\text{DH}}^{1-2}(r)\\ +\Delta G_{\text{np}}^{1-2}+\Delta G_{\text{rep}}^{1-2} \end{array}$$

where $\Delta G_{\text{el}}^{1-2}$ models long‐range electrostatic pair interactions, $G_{\text{edesolv}}^{1-2}$ models short‐range charge desolvation interactions and $\Delta G_{\text{DH}}^{1-2}(r)$ is a distance‐dependent Debye‐Hückel correction to the long‐range pair term, that accounts for the use of finite‐sized grids in this calculation. These first three terms approximate the Poisson‐Boltzmann electrostatic interaction free energy between solute pairs. The last two terms in eq. (A1) model the non‐polar interactions between solute pairs. $\Delta G_{\text{np}}^{1-2}$ is a non‐polar desolvation term that describes the change in the total solute‐solvent interface area as two solutes bind to each other, while $\Delta G_{\text{rep}}^{1-2}$ is a soft‐core repulsive term that prevents solutes from overlapping.

When simulating a system which contains a immobile surface, each of the interaction terms in eq. (A1), other than the long‐range electrostatic correction $\Delta G_{\text{DH}}^{1-2}(r)$, can be used to describe interactions with the surface. If a metal surface is present, SDA 7 includes additional interaction terms to account for the electrostatic interactions of solutes with the surface using a method of images charge model, for the desolvation of the metal surface, and for the van der Waals interactions between the mobile solute and the metal surface. These interactions form the basis of the ProMetCS force field, described by Kokh et al.^10^

### Electrostatic interactions

Electrostatic interactions, because of their long range, are a driving component in the dynamics of biomacromolecular association. The electrostatic solvation free energy of a charged solute in solution can be obtained by first solving the Poisson–Boltzmann equation to obtain the electrostatic potential induced in a polarisable dielectric medium by the charge density of the solute, then calculating the work required to charge the solute within this induced potential. The electrostatic interaction free energy between a pair of solutes separated at a distance from each other can be calculated as the difference between the electrostatic solvation free energy of the combined two solute system and the individual solutes. To avoid this expensive calculation at each step of the simulated trajectory, SDA employs the Effective Charge Model (ECM), first described by Gabdoulline and Wade,^28^ to approximate the Poisson‐Boltzmann derived electrostatic interaction free energy.

#### Effective charges and long‐range electrostatic interactions

In the Effective Charge Model (ECM),^28^ the electrostatic potential surrounding each solute, in isolation within a polarisable dielectric medium, is calculated prior to simulation, by solution of the Poisson–Boltzmann equation, using a set of partial atomic charges to represent the solute charge density and suitable solute interior and solvent dielectric constants. A set of effective solute charges are then generated, which are fitted such that they, in a uniform dielectric, reproduce the computed electrostatic potential in a shell around the solute. During simulations, the long‐range electrostatic interaction free energy between a pair of solutes $\Delta G_{\text{el}}^{1-2}$ is calculated from the interaction between the effective charges on one solute with the electrostatic potential field surrounding the other, according to

(A2) $$\Delta G_{\text{el}}^{1-2}=\frac{1}{2}\sum_{i_{1}}q_{i_{1}}\;\Phi_{{\text{el}}_{2}}(r_{i_{1}})+\frac{1}{2}\sum_{i_{2}}q_{i_{2}}\;\Phi_{{\text{el}}_{1}}(r_{i_{2}})$$

where $q_{i_{n}}$ is an effective charge on solute *n* and $\Phi_{{\text{el}}_{m}}(r_{i_{n}})$ is the electrostatic potential of solute *m*, at the position of effective charge $q_{i_{n}}$ on solute *n*. The $\frac{1}{2}$ factors are present to prevent double counting of the interaction.

#### Short‐range electrostatic desolvation

As a pair of solutes approach each other to form a binding interface, the exclusion of the high dielectric solvent from this interface results in the desolvation of surface‐lying charges. This results in an unfavorable contribution to the binding free energy of the two solutes. The long‐range electrostatic term described above is unable to account for this effect, so in SDA this is modeled separately, as the interaction between effective charges on one solute and the electrostatic desolvation potential of the other solute,^42^ according to

(A3) $$\Delta G_{\text{edesolv}}^{1-2}=\sum_{i_{1}}q_{i_{1}}^{2}\;\Phi_{{\text{edesolv}}_{2}}(r_{i_{1}})+\sum_{i_{2}}q_{i_{2}}^{2}\;\Phi_{{\text{edesolv}}_{1}}(r_{i_{2}})$$

where $\Phi_{{\text{edesolv}}_{m}}(r_{i_{n}})$, the electrostatic desolvation potential of solute *m* at the position of effective charge $q_{i_{n}}$ on solute *n*, describes the effect of the reduction in the dielectric constant at this point. The potential at point **r** surrounding a solute is given by

(A4) $$\Phi_{\text{edesolv}}(r)=\alpha \frac{\epsilon_{s}-\epsilon_{p}}{\epsilon_{s}(2\epsilon_{s}+\epsilon_{p})}\sum_{j}a_{j}^{3}\frac{{\left(1+\kappa r_{j}\right)}^{2}}{r_{j}^{4}}e^{-2\kappa r_{j}}$$

where *α* is an empirical scaling parameter, *ϵ*_*s*_ and *ϵ*_*p*_ are the solvent and solute interior dielectric constants, respectively, *κ* is the inverse of the Debye length, which is dependent on the ionic strength of the solvent. The sum runs over all solute atoms *j* of radius *a*_*j*_. The parameter *α* is used to scale the interaction.

#### Long‐range Debye–Hückel electrostatic correction

The use of finite sized grids to model long‐range electrostatic interactions can introduce errors due to the truncation of the potential at the grid boundary. To minimize such errors, it is necessary to use grids that are large enough such that this truncation error remains small. In low ionic strength media, where the low charge shielding of the solvent means that the electrostatic potential surrounding a solute decays slowly with distance, this can require grid sizes that demand significant memory resources during calculations.

SDA 7 includes a Debye‐Hückel electrostatic correction term^32^ that allows for the truncation of grid‐based electrostatic interactions, while modeling longer‐range interactions with a distance‐dependent term. At separations *r* where the electrostatic potentials surrounding two solutes can be approximated as being spherically symmetric, the interaction between the solutes can be modeled as that between a pair of Debye‐Hückel spheres of net charges *z*_1_ and *z*_2_, and radii *a*_1_ and *a*_2_, using

(A5) $$\Delta G_{\text{DH}}^{1-2}(r)=\frac{z_{1}z_{2}e_{l}^{2}e^{-\kappa (r-(a_{1}+a_{2}))}}{4\pi \epsilon_{0}\epsilon_{s}r(1+\kappa (a_{1}+a_{2}))}$$

where *e*_*l*_ is the elementary charge, *ϵ*_0_ is the vacuum permittivity, *ϵ*_*s*_ the dielectric constant of the solvent, and *κ* is the inverse of the Debye length. The net charge on each solute is taken as the sum of the effective charges on that solute, while the radius is the Stokes hydrodynamic radius.

To smooth the transition between the grid‐based distance‐dependent electrostatic calculations, the electrostatic grids are made spherical. When the atoms of one solute straddle the spherical grid boundary of a second, a hybrid calculation is performed, with interactions due to the effective charges lying within the boundary modeled with the full grid treatment, and the net charge used in the Debye‐Hückel correction reduced to account for only those charges lying outside the boundary.

### Nonpolar interactions

#### Nonpolar desolvation

Placing a solute in solution requires that a cavity of suitable size is created within the solvent, an energetically unfavorable process. As two solutes form a binding interface with each other, the total solute–solvent interface is reduced, resulting in an increased binding affinity. In SDA 7, this interaction is assumed to be proportional to the solvent accessible surface area of a solute that is occluded by the interacting solute, as described by Gabdoulline and Wade.^9^ It is calculated as

(A6) $$\Delta G_{\text{np}}^{1-2}=\sum_{i_{1}}{\text{SASA}}_{i_{1}}\;\Phi_{{\text{np}}_{2}}(r_{i_{1}})+\sum_{i_{2}}{\text{SASA}}_{i_{2}}\;\Phi_{{\text{np}}_{1}}(r_{i_{2}})$$

where $\Phi_{{\text{np}}_{m}}(r_{i_{n}})$ is the non‐polar burial potential of solute *m* at the position of surface‐lying atom *i*_*n*_ of solute *n*, and ${\text{SASA}}_{i_{n}}$ is the solvent accessible surface of atom *i*_*n*_. The non‐polar burial potential at a position **r** surrounding a protein is given by

(A7) $$\Phi_{\text{np}}(r)=\beta c\{\begin{array}{cc} 1 & r_{\text{min}}<a\\ \frac{b-r_{\text{min}}}{b-a} & a<r_{\text{min}}<b\\ 0 & r_{\text{min}}>b \end{array}$$

where factor *β* is the constant of proportionality between buried area and non‐polar desolvation energy, *c* is a factor that prevents double counting of the buried area when calculating the sum in eq. (A6), parameters *a* and *b* define the maximum distance an interacting solute atom can lie from the molecular surface, while being totally occluded by it, and the minimum distance at which its solvation is not affected, respectively. The distance $r_{\text{min}}$ represents the minimum distance from **r** to the molecular surface of the solute.

Inclusion of the non‐polar desolvation term was found important for the computation of protein‐protein electron transfer rates with SDA.^9^

#### Solute Repulsion

Repulsive interactions that prevent the overlap of solutes are modeled in two ways in SDA. In bimolecular simulations, overlaps are prevented by the use of excluded volume grids. The excluded volume grid of a solute is defined by the molecular surface of that solute. If a BD move places an atom of one solute within the excluded volume of another solute, the move is repeated with a different random displacement until a move is made that results in no overlap.

A soft‐core repulsive interaction term was introduced^11^ to remove the need to test for excluded volume violations in many‐molecule simulations. The repulsive interaction is given by

(A8) $$\Delta G_{\text{rep}}^{1-2}=\sum_{i_{1}}E_{{\text{rep}}_{2}}(r_{i_{1}})+\sum_{i_{2}}E_{{\text{rep}}_{1}}(r_{i_{2}})$$

where the sums run over all solvent accessible atoms in each solute and $E_{rep_{m}}$ is the repulsive interaction potential which tends to a large but finite positive value at zero separation and decays to zero at large separations. The potential at a point **r** around a solute is calculated according to

(A9) $$E_{\text{rep}}(r)=\gamma \sum_{i}\frac{1}{{\left(\frac{a_{i}}{\sigma }\right)}^{\text{nexp}}+|r-r_{i}{|}^{\text{nexp}}}$$

where $r_{i}$ is the position of solvent accessible surface atom *i* of the solute, *a*_*i*_ is its radius, *γ* is a parameter that defines the magnitude of the function, parameter *σ* defines the smoothness of the potential and nexp its distance decay rate.

### Interactions of mobile solutes with metal surfaces

While biomacromolecular association may be predominantly controlled by long‐range electrostatic interactions, the association of biomacromolecules to uncharged metal surfaces can be controlled by polarisation effects that occur at a much shorter range. Kokh et al^10^ developed the ProMetCS force field to account for these effects in BD simulations of proteins in the presence of a gold surface, and this force field is available in SDA 7. The interaction terms used in the force field are described below.

#### Electrostatic Interactions of Solutes with Conducting Surfaces

SDA 7 represents the electrostatic interaction of a solute *S* with a conductive metal surface *M* using a method of images charge model, which is implemented using an effective charge approximation analogous to the solute‐solute electrostatic model. In this, the solute‐surface electrostatic interaction is defined as an interaction between the solute effective charges *q*_*i*_, immersed in a uniform high‐dielectric solvent medium, with the electrostatic potential of the solute image reflected in the plane of the surface. The interaction is given by

(A10) $$\Delta G_{{\text{el}}_{\text{im}}}^{M-S}=\sum_{i}\Phi_{{\text{el}}_{\text{im}}}(r_{i})q_{i}$$

where the electrostatic potential of the solute image is $\Phi_{{\text{el}}_{\text{im}}}(r_{i})\equiv -\Phi_{{\text{el}}_{S}}(r_{j})$, and $r_{i}\equiv (x_{i},y_{i},z_{i}+2z_{CG})$ and $r_{j}\equiv (x_{i},y_{i},-z_{i}-2z_{CG})$ are vectors from the geometric centres of the image and real protein, respectively, to an effective charge *i*, and *z*_*CG*_ is the height of the solute centre above the metal surface. As a solute approaches distances close to metal surface, charged atoms on the surface of the solute become desolvated, and this is accounted for with an electrostatic desolvation term analogous to eq. (A3).

At small solute‐surface separations, the low‐dielectric cavities of the solute and the metal begin to merge (i.e. the solute partially or completely replaces the hydration shell of the metal at the solute‐metal interface). This produces a highly favorable binding interaction. A short‐range electrostatic correction term is employed to model this strong electrostatic binding. It is calculated for all effective charges at heights *z* < 5.5 Å above the surface, as a Coulomb interaction with their images in a low‐dielectric environment. For this a variable dielectric constant is introduced as $\epsilon (z)=4.0+0.8z^{2}+\text{exp}(\frac{z}{0.385}-10.4)$, where *z* is in Å, (see details of parameterisation procedure in Ref^10^). The short‐range analytical electrostatic correction term replaces the standard electrostatic potential (for the dielectric constant of solvent *ϵ*_*s*_) for the corresponding image charges. The short‐range analytical correction $\Delta G_{\text{corr}}^{M-S}$ is calculated as

(A11) $$\Delta G_{\text{corr}}^{M-S}=\sum_{i}\sum_{j}\frac{q_{i}q_{j}}{2r_{ij}(4\pi \epsilon_{0})}\left(\frac{1}{\epsilon_{s}}-\frac{2}{\epsilon (z_{i}+z_{j})}\right)$$

where *r*_*ij*_ is the distance between a real effective charge *q*_*i*_ and image charge *q*_*j*_, and *z*_*i*_ and *z*_*j*_ are the distances between effective charges *i* and *j* and the plane of the surface.

The implemented approximation for short‐range electrostatic interactions based on the values of effective charges should be treated with caution if the interactions are very strong, ie if a very low ionic strength solution or highly charged mobile solutes (for example, DNA) are used. In these cases, the charges used in the short‐range electrostatic term can be scaled using the input parameter *correction_image_charge*.

#### Metal Surface Desolvation

As a solute *S* approaches and adsorbs to a metal surface *M*, it must displace solvent molecules from the hydration layers of the metal. The effects of this displacement can be modeled using a metal desolvation potential

(A12) $$\Delta G_{\text{metd}}^{M-S}=\sum_{i}\Phi_{\text{metd}}(z_{i})S_{i}^{\text{desolv}}$$

where $S_{i}^{\text{desolv}}$ is the area of the metal surface desolvated by a solute atom *i* and $\Phi_{\text{metd}}(z)$ is the metal desolvation potential at a height *z* above the metal surface, which is given by

(A13) $$\Phi_{\text{metd}}(z_{i})=\{\begin{array}{cc} \Phi_{\text{metd}}^{0} & z_{i}\leq Z_{\text{adw}}\\ \Phi_{\text{metd}}^{0}\text{exp}(-(z_{i}-Z_{\text{adw}}/\gamma )) & Z_{\text{adw}}<z_{i}<Z_{\text{max}}\\ 0 & z_{i}>Z_{\text{max}} \end{array}$$

where $\Phi_{\text{metd}}^{0}$ is the desolvation potential of the first hydration layer, $Z_{\text{adw}}$ is the height of the first hydration layer above the metal surface, and $Z_{\text{max}}$ is the maximum height a solute can be above the metal surface, while still affecting its solvation.

#### Lennard–Jones interactions

Kokh et al.^10^ introduced a Lennard‐Jones interaction term to model the van der Waals and weak chemical interactions between solutes and metal surfaces, as a component of the ProMetCS force field. In contrast to the other force field terms, interaction potential grids are calculated for each solute atom type *t*_*S*_. The total Lennard‐Jones interaction energy between a solute *S* and metal surface *M* is given by

(A14) $$\Delta G_{\text{LJ}}^{M-S}=\sum_{t_{M}}\sum_{i_{M}}\Phi_{{\text{LJ}}_{S}}^{t_{M}}(r_{i_{M}})$$

where the potential $\Phi_{{\text{LJ}}_{S}}^{t_{M}}(r_{i_{M}})$, centred on solute *S*, describes the Lennard‐Jones interaction of a probe atom of type *t*_*M*_ at the position of atom *i*_*M*_ of this type on the interacting surface. The potential $\Phi_{\text{LJ}}^{t_{M}}(r)$ that acts on a probe atom of type *t*_*M*_ at a position **r** from the solute centre, is given by

(A15) $$\Phi_{\text{LJ}}^{t_{M}}(r)=\sum_{t_{M}}\sum_{i_{S}}4\epsilon_{t_{M}t_{S}}\left\{{\left(\frac{\sigma_{t_{M}t_{S}}}{r}\right)}^{12}-{\left(\frac{\sigma_{t_{M}t_{S}}}{r}\right)}^{6}\right\}$$

where the first sum runs over the atom types *t*_*M*_ on the metal surface, the second sum runs over atoms *i*_*S*_ of the solute, *t*_*S*_ is the atom type of solute atom *i*_*S*_, *r* is the distance from the centre of the solute to the point **r**, and $\epsilon_{t_{M}t_{S}}$ and $\sigma_{t_{M}t_{S}}$ are Lennard‐Jones parameters describing the interaction between atoms of type *t*_*M*_ and *t*_*S*_.

In ProMetCS,^10^ eqs. (A14) and (A15) have been been parameterized for the interaction of proteins with gold surfaces by fitting to the all‐atom GolP force field of Iori et al.^43^
