# Supplementary material for: SDA 7: A modular and parallel implementation of the simulation of diffusional association software
Source: J Comput Chem. 2015 Jun 29;36(21):1631–45. doi: 10.1002/jcc.23971 (PMC4755232; doi:10.1002/jcc.23971)
Supplement: Supplementary file 1 — Supporting Information [file JCC-36-1631-s001.pdf]

Supporting Information for SDA 7: A modular and parallel implementation of the Simulation of Diffusional Association software.

Michael Martinez[†] Neil J. Bruce[†*] Julia Romanowska[†**]
Daria B. Kokh[†] Musa Ozboyaci[†‡] Xiaofeng Yu[†§]
Mehmet Ali Öztürk[†§] Stefan Richter[†] Rebecca C. Wade[†¶‖*]

[†]Molecular and Cellular Modeling Group, Heidelberg Institute for Theoretical Studies (HITS), Schloss-Wolfsbrunnenweg 35, 69118 Heidelberg, Germany

[‡]Heidelberg Graduate School of Mathematical and Computational Methods for the Sciences (HGS MathComp), Im Neuenheimer Feld 368, 69120 Heidelberg, Germany.

[§]Hartmut Hoffmann-Berling International Graduate School of Molecular and Cellular Biology (HBIGS), Im Neuenheimer Feld 501, 69120 Heidelberg, Germany.

[¶]Zentrum für Molekulare Biologie der Universität Heidelberg (ZMBH), DKFZ-ZMBH Alliance, Im Neuenheimer Feld 282, 69120 Heidelberg, Germany.

[‖]Interdisciplinary Center for Scientific Computing (IWR), Heidelberg University, Im Neuenheimer Feld 368, 69120 Heidelberg, Germany.

[*]Corresponding author.

[**]Current address: Department of Global Public Health and Primary Care, University of Bergen, Kalfarveien 31, 5018 Bergen, Norway.

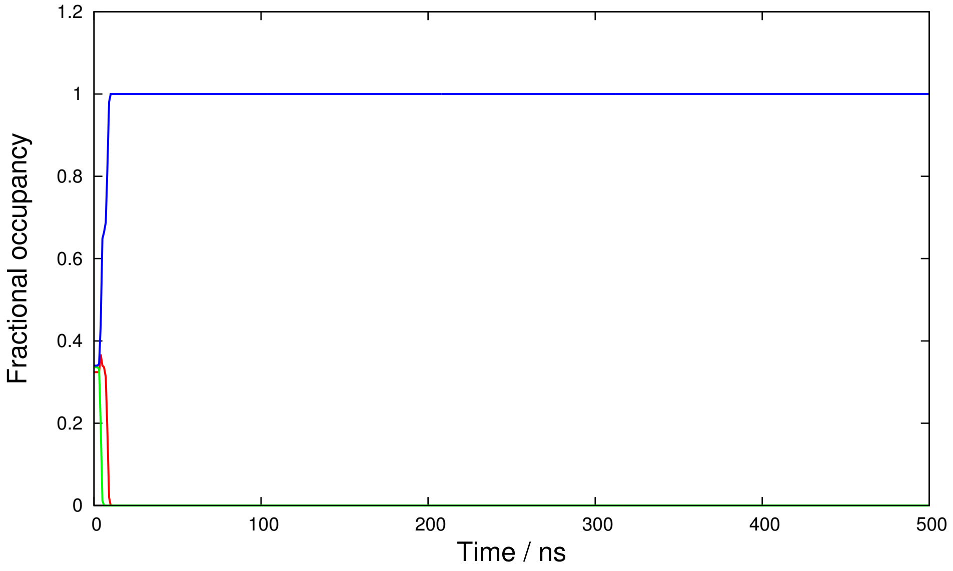

Figure S1: Fractional occupancies of the pH 3 (red), pH 6 (green) and pH 9 (blue) protonation states of HEWL during a 500 ns BD simulation of 256 HEWL molecules, with trial transitions between states accepted according to the minimum energy criterion.

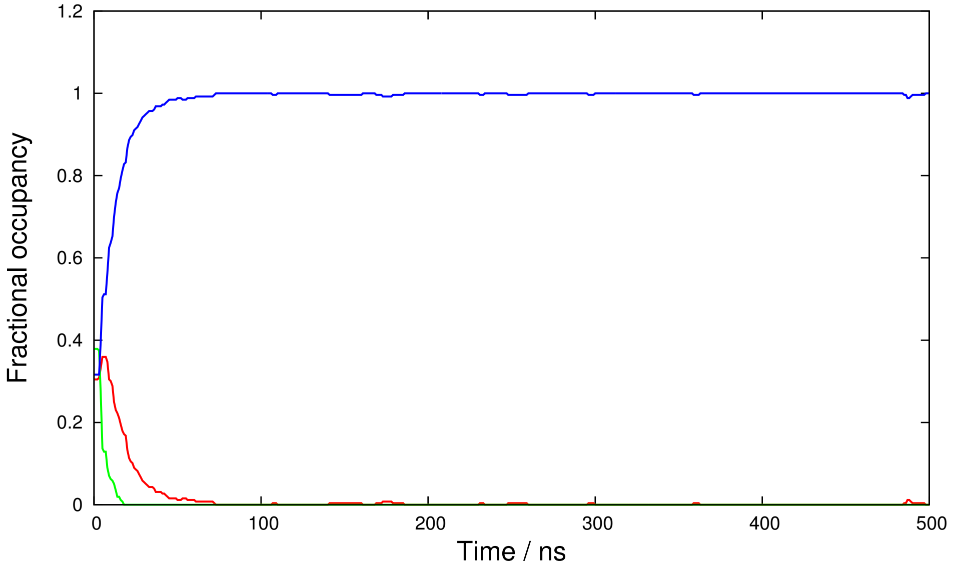

Figure S2: Fractional occupancies of the pH 3 (red), pH 6 (green) and pH 9 (blue) protonation states of HEWL during a 500 ns BD simulation of 256 HEWL molecules, with trial transitions between states accepted according to the Metropolis criterion.
